# Clinical Relevance of Transforming Growth Factor-β1, Interleukin-6 and Haptoglobin for Prediction of Obesity Complications in Prepubertal Egyptian Children

**DOI:** 10.3889/oamjms.2015.017

**Published:** 2015-01-22

**Authors:** Inas R. El-Alameey, Nevein N. Fadl, Enas R. Abdel Hameed, Lobna S. Sherif, Hanaa H. Ahmed

**Affiliations:** 1 *Child Health Department, National Research Centre, Cairo, Egypt*; 2 *Medical Physiology Department, National Research Centre, Cairo, Egypt*; 3 *Hormones Department, National Research Centre, Cairo, Egypt*

**Keywords:** Obesity Complications, Egyptian Children, Prepubertal, prediction, transforming growth factor-β1, interleukin-6, haptoglobin, clinical relevance

## Abstract

**BACKGROUND:**

The rate of obesity is increasing throughout the world. Obesity in adults’ research is characterized by chronic inflammation, associated with type 2 Diabetes and cardiovascular risk. The degree to which these changes occur in childhood obesity is not fully defined.

**AIM:**

This study was designed to explore the relation between circulating levels of pro-inflammatory cytokines, and obesity.

**PATIENTS AND METHODS:**

This cross sectional case control study was carried out in 50 randomly selected pre-pubertal overweight and obese children compared with fifty apparently healthy children of matched age and sex. Serum levels of transforming growth factor-β1, interleukin-6, and haptoglobin were quantified by ELISA technique.

**RESULTS:**

ANOVA test followed by Post Hoc test showed highly significant increase in the serum levels of the transforming growth factor-β1, interleukin-6 and haptoglobin among obese children compared to overweight and healthy children respectively. The body weight, BMI and BMI z-score were significantly positively correlated with serum levels of the three pro-inflammatory cytokines. Serum levels of interleukin-6, and haptoglobin were found to be strong predictors of complications in severe obesity by linear regression analysis.

**CONCLUSIONS:**

Obesity is associated with chronic low-grade inflammation. High levels of interleukin-6 and haptoglobin are considered to be early biomarkers of inflammation associated with severe obesity with subsequent cardiovascular and type 2 diabetes risk.

## Introduction

Obesity is a serious and progressively increasing public health problem that has reached epidemic proportions with an increasing worldwide prevalence [1]. The prevalence of childhood and adolescent obesity has tripled dramatically over the last 20–30 years in developed countries, with recent estimates to be ~17% in the United States [2]. With increasing epidemics of obesity all over the world, research in adult populations have established the link between elevated inflammatory markers and impaired health. Obesity is found to be associated with low-grade inflammatory process characterized by the increase in circulating levels of pro-inflammatory cytokines such as transforming growth factor-β1, interleukin-6 and acute-phase protein (haptoglobin) in healthy obese adults [3-4].

Haptoglobin is an adiposity and inflammatory marker that involved in the liver acute phase response to inflammation [5]. The most important functions of haptoglobin are to bind free hemoglobin, thus preventing its oxidant activity [6]. Interleukin-6 has been recently proposed to play a central role in the link between obesity, inflammation and coronary heart diseases [7-8]. Bastard et al., have suggested that IL-6 could be involved in insulin resistance and its complications [9]. About 15 to 30 % of circulating IL-6 levels derives from adipose tissue production in the absence of an acute inflammation [10]. It regulates energy homeostasis and inflammation; it is capable of suppressing lipoprotein lipase activity, and controls appetite and energy intake at hypothalamic level [11-12].

TGF-β_1_ is a new multifunctional cytokine that is produced by a variety of cells. TGF-β_1_ activation is closely associated with damage the intima media and lining endothelium of blood vessels through the stimulation of atherosclerotic lesions that is closely associated with the development of CVD, including hypertension [13], cardiac hypertrophy and cardiac fibrosis [14] leading to heart failure, restenosis after coronary intervention and thrombosis [15]. In addition, inflammatory markers impact metabolic control by negatively influencing insulin sensitivity and glucose transport. Recent studies have shown a possible interaction between TGF-β_1_ and visceral fat obesity. It has been considered to play a central role in the development of metabolic syndrome in childhood which is predictive of adult metabolic syndrome, non-alcoholic fatty liver disease and type 2 Diabetes [16]. Furthermore, TGF-β_1_ is a potent initiator of proliferation of renal mesangial cells leading to chronic kidney disease and there are associations between serum level of TGF-β_1_ and risk factors for progression of clinically relevant renal disorders in humans [17].

Thus, prolonged exposure of obese individuals to these inflammatory markers may exponentially increase their risk in the development of vascular damage, cardiovascular disease, and hypertension [18]. In obese children, early identification of elevated inflammatory markers and development of physical activity interventions are important in attenuating the negative health consequences that are likely facilitated as they mature into adults [19]. The origin of inflammation during obesity and the underlying mechanisms that explain its occurrence are not still fully understood. To our knowledge, no data are available on the physiology, predictors and role of haptoglobin, and TGF-β_1_ in childhood obesity. Therefore, the focus of our interest was to evaluate predictors of obesity complications and the effect of childhood obesity on pro-inflammatory cytokines production as TGF-β_1_, IL-6, and haptoglobin, and to explore the relation between circulating levels of these cytokines and anthropometric measures among prepubertal overweight and obese Egyptian children.

## Subjects and Methods

### Subjects

This cross sectional case control study was conducted on fifty obese or overweight children randomly selected from new cases attending the Nutrition Outpatient Clinic of the National Research Center for nutritional management during 2014. Overweight & obese children were identified using BMI for age and sex charts according to standardized methods of the World Health Organization (WHO). Obesity is defined as BMI and weight for age more than 2 SDs above the median value of the WHO International Growth References, and BMI z-score more than 1.66 SDs above the WHO age and sex specific mean for overweight [20]. Inclusion criteria for selection included pre-pubertal school overweight & obese children with simple obesity. Exclusion criteria included children having obesity syndromes, or taking medications associated with weight change as glucocorticoid therapy. None of the participants had chronic illness as cardiovascular or endocrine disease as type I or II diabetes, hypothyroidism, and Cushing’s disease. Age and sex matched fifty apparently healthy non obese children have been selected as the control group. They recruited from the outpatient’ clinic at National Research Center. Ethical approval was obtained from the Medical Ethical Committee of the National Research Center. Written informed consent was obtained from the parents after explanation of the aim of the study and its possible benefits for identifying the cause of the obesity of their children and other children who have the same condition.

## Methods

All children in the study were subjected to full personal, past history for systemic diseases, and drug administration as corticosteroids. Each child was subjected to a complete physical examination and anthropometric measures. All measurements were made according to techniques described in the Anthropometric Standardization Reference Manual [20]. Children were weighed (in kg) using a calibrated Seca scale to the nearest 0.1 kg (Seca, Hamburg, Germany), while height (in cm) was measured using a Seca 225 stadiometer to the nearest 0.1 cm with the children dressed in minimal clothes, and without shoes. Each measurement was taken as the mean of three consecutive readings following the recommendations of the International Biological program [21].

For all children, growth parameters including BMI (kg/m^2^), and BMI z-scores were computed using WHO growth standards [22] with the help of Anthro-Program of PC, and online software (http://www.WHO.gov/epiinfo). Body mass index (BMI) was calculated according to the known formula as the body weight in kilograms divided by the square of height in meters (kg/m^2^) to classify overweight and obese children. Assessment of BMI was done using categories reported by the world health organization child growth charts standards for age and sex. A body mass index below 18.5 means that the child is underweight, 18.5 to 24.9 means that the child fall in the normal range, 25.0 to 29.9 means child is overweight and a score of 30.0 and higher means the child is obese.

Venous blood samples were drawn by venipuncture after 12 hours overnight fasting. Blood samples were immediately centrifuged, and serum was frozen at -80°C until assay. Serum IL-6 was determined by Sandwich enzyme linked immunosorbent assay (ELISA) as the method using a Human IL-6 assay kit provided from Immundiagnostik AG, Bensheim, Germany, according to Bauer et al., [23]. Quantitative determination of the transforming growth factor-β_1_ (TGF-β_1_) in serum samples was performed by Sandwich enzyme linked immunosorbent assay (ELISA) method using Assay Max Human transforming growth factor-β_1_ ELISA Kit from Assay Pro Co., Millipore, St. Charles, MO, (USA), according to Kropf et al., [24]. This assay employs a quantitative sandwich enzyme immunoassay technique that measures TGF-β_1_ in less than 5 hours. Serum haptoglobin was assayed by Sandwich enzyme linked immunosorbent assay (ELISA) as the method using a Human haptoglobin assay kit purchased from Assay Pro Co. (USA) according to Van Vlierberghe et al., [25]. This assay measures haptoglobin in less than 2 hours.

### Statistical analysis

Statistical analysis was performed using the SPSS statistical package software for windows version 21 (SSPS Inc, Pennsylvania, and USA). Parametric variables are expressed as the mean ± SD. Differences between parametric variables among the obese and overweight versus control groups were evaluated using 2-tailed unpaired t-test. The comparison between groups was performed with one way analysis of variance (ANOVA) with Post HOC. Pearson’s correlation coefficients were used to evaluate correlations between the data exhibiting parametric distribution. Linear logistic regression analysis was performed to examine the relationship between serum levels of TGF-β_1_, interleukin-6, and haptoglobin with anthropometric measures. P value <0.05 was considered significant difference and p<0.001 was considered highly significant difference.

## Results

A total of fifty overweight and obese children aged between 5 to 12 years (mean 9.28 ± 1.96 years) were studied. Out of them, 30 children were obese and 20 children were overweight. The comparison of the anthropometric measures and pro-inflammatory cytokines of obese group and overweight group versus control group by ANOVA test are shown in Table 1. ANOVA test followed by Post Hoc test showed statistically highly significant increase in the serum levels of TGF-β_1_, interleukin-6, and haptoglobin in obese children compared to overweight children, and healthy controls respectively (P<0.001, in all).

**Table 1:** The anthropometric measures, and pro-inflammatory cytokines of obese group and overweight group versus control group.

Variables	Control (group III) N=50	Obese group (group I) N=30	Overweight (group II) N=20	F	P-value
Mean± SD	Mean± SD	Mean± SD
Weight (kg)	33.64±6.61	50.8±10.39	46.55±7.23	17.16	<0.001**
Height (cm)	140±12.08	121.07±10.37	128.7±9.7	29.63	<0.001**
BMI (kg/m2)	16.84±1.01	34.81±4.38	27.96±1.71	463.97	<0.001**
BMI z-score	0.12±0.49	2.83±0.44	2.34±0.42	367.33	<0.001**
Serum haptoglobin (μg/ml)	2.06±0.76	7.84±1.57	4.69±1.01	253.89	<0.001**
Serum TGF- β1 (ng/ml)	23.76±4.62	45.38±5.14	40.31± 5.22	220.41	<0.001**
Serum IL-6 (pg/mL)	1.13±0.35	12.29± 0.97	8.75±1.55	1549.29	<0.001**

*Significant difference at p<0.05,

** highly significant difference at p<0.001.

*** ANOVA.

Serum levels of TGF- β_1_, IL-6 and haptoglobin were significantly positively correlated with the body weight, BMI (kg/m^2^) and BMI z-score of obese/overweight cases, respectively (P<0.001, in all), while serum levels of IL-6 and haptoglobin were significantly inversely correlated with their height. Serum level of TGF- β_1_ was significantly positively correlated with serum levels of IL-6 and haptoglobin in obese and overweight children. Correlations between anthropometric measurements and pro-inflammatory cytokines in obese/overweight group are shown in Table 2.

**Table 2:** Correlations between anthropometric measurements and pro-inflammatory cytokines in obese / overweight group.

Variables		Weight	Height	BMI	BMI z-score	Serum hapto-globin	Serum TGF-β1
Serum Haptoglobin (μg/ml)	Pearson Correlation	0.435**	-0.293*	0.876**	0.575**	1	0.560**

	Sig. (2-tailed)	0.002	0.039	.000	0.000		0.000

Serum TGF- β1 (ng/ml)	Pearson Correlation	0.185**	-0.229	0.564**	0.391**	0.560**	1

	Sig. (2-tailed)	0.001	0.109	0.000	0.005	0.000	

Serum IL-6 (pg/mL)	Pearson Correlation	0.383**	-0.292*	0.809**	0.512**	0.831**	0.558**

	Sig. (2-tailed)	0.006	0.04	0.000	0.000	0.000	0.000

* Significant difference at p<0.05,

** highly significant difference at p<0.001.

In overweight children, serum level of IL-6 was significantly positively correlated with BMI (r = 0.593, P = 0.006), and serum level of haptoglobin (r = 0.458, P = 0.042), and no significant correlation was found between serum levels of IL-6 and TGF-β_1_ (P > 0.05). Correlations between serum level of IL-6 and BMI, serum levels of haptoglobin and TGF-β_1_ of overweight children are shown in Table 3.

**Table 3:** Correlations between serum level of IL-6 and body mass index, serum levels of haptoglobin, and TGF- β1 in overweight group.

Variable		BMI	Serum Haptoglobin	Serum TGF β1
Serum IL-6 (pg/mL)	Pearson Correlation	0.593**	0.458*	0.204

	Sig. (2-tailed)	0.006	0.042	0.389

* Significant difference at p<0.05,

** highly significant difference at p<0.001.

To confirm the previous correlations, linear regression analysis was done showing the association between the different biomarkers studied and body mass index. Serum levels of IL-6 and haptoglobin were strong predictors in the prediction of obesity complications (P < 0.05) as shown in Table 4.

**Table 4:** Linear logistic regression between body mass index and serum pro-inflammatory cytokines in obese / overweight group.

Variables	Unstandardized coefficients		Standardized coefficients	t	P-value
	B	Std. Error	Beta		
Serum haptoglobin (μg/ml)	1.500	0.291	0.635	5.154	0.000**
Serum TGF-β1(ng/ml)	0.065	0.071	0.075	0.912	0.367
Serum IL-6 (pg/mL)	0.548	0.282	0.239	1.943	0.048*

Dependent variable: BMI.

* Significant difference at p<0.05,

** highly significant difference at p<0.001.

## Discussion

The National Health and Nutrition Examination Survey (NHANES) reported that the prevalence of obesity is increasing in all pediatric age groups, in both sexes, and in various ethnic and racial groups [26]. Higher percentages of obesity among Egyptian school children were reported [27- 28].

Studies in mice and adult humans have associated elevated levels of pro-inflammatory cytokines with metabolic, cardiovascular diseases (CVD) and cardiac death [29-32]. It has not yet been shown in prepubertal children how pro-inflammatory cytokines and BMI are interrelated. To our best knowledge, this current study is considered to be the first clinical study that was carried out to evaluate the predictors of obesity complications and the effect of childhood obesity on pro-inflammatory cytokines production as TGF-β_1_, IL-6, and haptoglobin among prepubertal overweight and obese Egyptian children.

IL-6 is a pro-inflammatory cytokine that plays important roles in acute phase reactions, and inflammation. It is produced by many cell types, and adipose tissue, that enhanced in obesity. It has been proposed as a screening tool to assess the risk for metabolic syndrome and coronary heart diseases in youth [33]. In our present study, serum levels of IL-6 showed statistically highly significant increase in obese children compared to overweight children and healthy controls respectively (P<0.001), and its serum level was significantly positively correlated with the body weight, BMI, and BMI z-score respectively (P<0.001). This is in the agreement with Steene et al., who reported that, IL-6 correlates with anthropometric markers and body composition in childhood. A positive association between adipocyte diameter and IL-6 levels has been described in humans [34]. It means there is a strong relationship between BMI and circulating levels of IL-6.

Haptoglobin levels had been reported to be associated with obesity [35], and evaluated for its role in inflammation in subjects with increased BMI. The increased synthesis of haptoglobin has been thought to be a consequence of IL-6, secreted by adipocytes [36]. In our present study, serum level of haptoglobin showed statistically highly significant increase in obese children (F=253.89, P<0.001) compared to overweight children and healthy controls respectively, and its serum level was significantly positively correlated with the body weight, BMI, and BMI z-score, respectively (P<0.001). It means, there is a positive association between BMI and circulating levels of haptoglobin.

TGF-β_1_ was suggested as an early biomarker of inflammation and CVD risk. Although some research report increased TGF-β_1_ level in adipose tissue in adults and mice [37], there is limited data in the literature concerning TGF-β_1_ concentration in obese humans. In the present study, serum levels of TGF-β_1_ showed statistically highly significant increase in obese (F=220.41, P<0.001) compared to overweight children and healthy controls respectively. This is in the agreement with Romano et al. [38] who reported higher serum TGF-β_1_ levels in obese women. On the contrary, other studies in obese adults showed decreased levels of TGF-β_1_ as in a study by Kinik et al., [39] who determined that obese children had lower TGF-β_1_ levels compared to leans, however, in a study by Kanra et al. [40] who reported the lack that the lack of an association between TGF-β_1_ polymorphisms and obesity in Turkish children.

In light of the current results, serum levels of TGF- β1 were found to be significantly positively correlated with the body weight, BMI, and BMI z- score. Our results is in the agreement with Fain et al., [37], who reported that, the TGF-β_1_ release was elevated in the presence of insulin and this may explain the positive correlation between BMI and TGF-β_1_ release that they saw in subcutaneous adipose tissue and the correlation between total release of TGF-β_1_ and body fat content. Our present study is not in the agreement with a study by Yener et al., [41] who reported that, serum TGF-β_1_ levels were lower and inversely correlated with body mass index (BMI) and waist circumference, and Kinik et al., [39] who reported that obese children had lower TGF-β_1_ levels compared to leans and the lower TGF-β1 levels were not correlated with lipids, insulin resistance and BMI.

IL-6 as a pro-inflammatory cytokine is a main stimulator of the production of most acute phase protein such as haptoglobin [36], and TGF-β_1_ [42]. This is in favor to our results in which serum level of IL-6 is significantly positively correlated with serum levels of TGF-β_1_ and haptoglobin. Moreover, in recent years, Aihara et al., [12], observed that, increasing expression of TGF-β_1_ from fatty tissue affects its serum level and hence may stimulate expression of the other cytokines. This could be explained why serum level of TGF-β_1_ was positively correlated with serum levels of IL-6 and haptoglobin among obese children in our present study.

Interestingly, linear regression analysis revealed that serum levels of IL-6 and haptoglobin play a significant role in the pathogenesis of obesity. The high serum levels of IL-6 and haptoglobin are significant strong predictors of adiposity (p < 0.05 in both). It means there is a strong relationship between BMI and circulating levels of both IL-6 and haptoglobin. This is in the agreement with a study by Chiellini et al. [35] who reported that haptoglobin is an adiposity marker and its circulating levels being significantly related to the degree of obesity. A study by Ridker et al., also reported that IL-6 production by adipose tissue is enhanced in obesity. One of the main effects of IL-6 is the induction of hepatic CRP and haptoglobin production, which is known to be an independent, major risk marker of metabolic and cardiovascular complications [43].

In our present study, serum level of IL-6 was found to be significantly positively correlated with BMI and serum level of haptoglobin and no significant correlation was found between serum levels of IL-6, and serum level of TGF-β_1_ in overweight children. We found that the increase in the proinflammatory cytokines occurs early even in the overweight cases and the association between obesity and the inflammation markers is dependent on a degree of obesity. Our results are in conformity with Skinner et al. [44] who reported that, obesity is associated with increases in systemic pro-inflammatory markers. The association of obesity and low grade inflammation is strongly dependent on a degree of obesity.

In conclusion, our results provided clinical evidence that, obesity is associated with a state of chronic low-grade inflammation. Obese and overweight prepubertal children have elevated serum levels of IL-6, and haptoglobin and TGF-β1 which are known as markers of inflammation that may increase the cardiovascular and/or metabolic disease risk. Early identification of pro-inflammatory markers may help in identifying those at risk of cardiovascular events, and type 2 diabetes mellitus.

## References

[1] Finucane MM, Stevens GA, Cowan MJ (2011). National, regional, and global trends in body-mass index since. 1980: systematic analysis of health examination surveys and epidemiological studies with 960 country-years and 9.1 million participants. The Lancet.

[2] Ogden CL, Carroll MD, Kit BK, Flegal KM (2012). Prevalence of obesity and trends in body mass index among US children and adolescents 1999-2010. JAMA.

[3] Galcheva SV, Iotova VM, Yotov YT, Bernasconi S, Street ME (2011). Circulating proinflammatory peptides related to abdominal adiposity and cardiometabolic risk factors in healthy prepubertal children. Eur J Endocrinol.

[4] McMurray RG, Zaldivar F, Galassetti P (2007). Cellular immunity and inflammatory mediator responses to intense exercise in overweight children and adolescents. J Investig Med.

[5] aWang Y, Kinzie E, Berger FG, Lim SK, Baumann H (2001). Haptoglobin, an inflammation-inducible plasma protein. Redox Rep.

[6] Nielsen MJ, Moestrup SK (2009). Receptor targeting of hemoglobin mediated by the haptoglobins: roles beyond heme scavenging. Blood.

[7] Yudkin JS, Kumari M, Humphries SE, Mohamed-Ali V (2000). Inflammation, obesity, stress and coronary heart disease: is interleukin-6 the link?. Atherosclerosis.

[8] Tam CS, Garnett SP, Cowell CT (2010). IL-6, IL-8 and IL-10 levels in healthy weight and overweight children. Horm Res Paediatr.

[9] Bastard JP, Maachi M, Tran Van Nhieu J, Jardel C, Bruckert E, Grimaldi A, Robert JJ, Capeau J, Hainque B (2002). Adipose tissue IL-6 content correlates with resistance to insulin activation of glucose uptake both in vivo and in vitro. J Clin Endocrinol Metab.

[10] Sacheck J (2008). Pediatric obesity: an inflammatory condition?. Journal of Parenteral and Enteral Nutrition.

[11] Mohamed-Ali V, Goodrick S, Rawesh A, Katz DR, Miles JM, Yudkin JS, Klein S, Coppack SW (2007). Subcutaneous adipose tissue releases interleukin-6, but not tumor necrosis factor-alpha, in vivo. J Clin Endocrinol Metab.

[12] Maffeis C, Silvagni D, Bonadonna R, Grezzani A, Banzato C, Tatò L (2007). Fat cell size, insulin sensitivity, and inflammation in obese children. Journal of Pediatrics.

[13] Annes JP, Munger JS, Rifkin DB (2003). Making sense of latent TGF-βactivation. Journal of Cell Science.

[14] Aihara K, Ikeda Y, Yagi S, Akaike M, Matsumoto T (2011). Transforming Growth Factor-β1 as a Common Target Molecule for Development of Cardiovascular Diseases, Renal Insufficiency and Metabolic Syndrome. Cardio Res Pract.

[15] Leas KA (2010). Potential therapeutic targets for cardiac fibrosis: TGF-β, angiotensin, endothelin, CCN2, and PDGF, partners in fibroblast activation. Circulation Research.

[16] Morrison JA, Friedman LA, Wang P, Glueck CJ (2008). Metabolic syndrome in childhood predicts adult metabolic syndrome and type 2 diabetes mellitus 25 to 30 years later. J Pediatr.

[17] Suthanthiran M, Gerber LM, Schwartz JE (2009). Circulating transforming growth factor-β1 levels and the risk for kidney disease in African Americans. Kidney International.

[18] Herder C, Schneitler S, Rathmann W (2007). Low-grade inflammation, obesity, and insulin resistance in adolescents. J Clin Endocrinol Metab.

[19] Skinner AC, Steiner MJ, Henderson FW, Perrin EM (2010). Multiple markers of inflammation and weight status: Cross-sectional analyses throughout childhood. Pediatrics.

[20] Lohman T.G, Roche A.F, Martorell R (1988). Anthropometric standardization reference manual.

[21] Tanner JM, Hiernaux J, Jarman S, Weiner JS, Lourie JA, Weiner JS, Lourie SA (1969). Growth, and physique studies. Human biology: A guidance to fields methods.

[22] WHO, Anthro Plus for personal computers. Manual Software for assessing growth of the world’s children, and adolescents (2009). http://www.who.int/growthref/tools/en/.

[23] Bauer J, Herrmann F (1991). Interleukin-6 in clinical medicine. Ann Hematol.

[24] Kropf J, Schurek JO, Wollner A, Gressner AM (1997). Immunological measurement of transforming growth factor-beta I (TGF-beta 1) in blood;assay development and comparison. Clinical Chemistry.

[25] Van Vlierberghe H, Langlois M, Delanghe J (2004). Haptoglobin polymorphisms and iron homeostasis in health and in disease. Clin Chim Acta.

[26] National Center for Health Statistics, Health, United States: The prevalence of obesity with special features on socioeconomic status and health (2014).

[27] Hassan NE, EL-Ashry HH, Awad AH, El-Masry SA, Youssef MM, Sallam MM, Anwar M (2011). Adiponectin in obese children and its association with blood pressure and anthropometric markers. Medical Research Journal.

[28] El Derwi D, El Sherbiny N, Atta AH (2011). Exploring Fayoum (Upper Egypt) preparatory school students ‘and teachers’ attitude towards obesity as health risk. J Public Health Epidemiol.

[29] Arslan N, Erdur B, Aydin A (2010). Hormones and cytokines in childhood obesity. Indian Pediatr.

[30] Heriberto Rodríguez-Hernández, Luis E. Simental-Mendía, Gabriela Rodríguez-Ramírez, Miguel A (2013). Reyes-Romero: Obesity and Inflammation: Epidemiology, Risk Factors, and Markers of Inflammation. International Journal of Endocrinology.

[31] Gregor MF, Hotamisligil GS (2011). Inflammatory mechanisms in obesity. Annual Review of Immunology.

[32] Faloia E, Michetti G, de Robertis M, Luconi M P, Furlani G, Boscaro M (2012). Inflammation as a link between obesity and metabolic syndrome. Journal of Nutrition and Metabolism.

[33] Giannini C, de Giorgis T, Scarinci A, Ciampani M, Marcovecchio ML, Chiarelli F, Mohn A (2008). Obese related effects of inflammatory markers and insulin resistance on increased carotid intima media thickness in pre-pubertal children. Atherosclerosis.

[34] Steene J, Kolle E, Reseland JE, Anderssen SA, Andersen LB (2010). Waist circumference is related to low-grade inflammation in youth. Int J Pediatr Obes.

[35] Chiellini C, Santini F, Marsili A, Berti P, Bertacca A, Pelosini C (2004). Serum haptoglobin: a novel marker of adiposity in humans. J Clin Endocrinol Metab.

[36] Matthews VB, Allen TL, Risis S, Chan MH, Henstridge DC, Watson N (2010). Interleukin-6-deficient mice develop hepatic inflammation and systemic insulin resistance. Diabetologia.

[37] Fain JN, Tichansky DS, Madan AK (2005). Transforming growth factor -β1 release by human adipose tissue is enhanced in obesity. Metabolism.

[38] Romano M, Guagnano MT, Pacini G (2003). Association of inflammation markers with impaired insulin sensitivity and coagulative activation in obese healthy women. J Clin Endocrinol Metab.

[39] Kinik ST, Özbek N, Yüce M, Yazıcı AC, Verdi H, Ataç FB (2008). PAI-1 gene 4G/5G polymorphism, cytokine levels and their relations with metabolic parameters in obese children. Thromb Haemost.

[40] Kanra AR, Tulgar-Kinik S, Verdi H, Ataç FB, Yazici AC, Ozbek N (2011). Transforming growth factor-beta1 (509 C/T, 915 G/C, 869 T/C) polymorphisms are not related to obesity in Turkish children. Turk J Pediatr.

[41] Stolzman S, Bement MH (2012). Inflammatory Markers in Pediatric Obesity: Health and Physical Activity Implications. Infant, Child, & Adolescent Nutrition.

[42] Yener S, Demir T, Akinci B (2007). Transforming growth factor-beta 1 levels in women with prior history of gestational diabetes mellitus. Diabetes Res Clin Pract.

[43] Ridker PM (2003). Clinical application of C-reactive protein for cardiovascular disease detection and prevention. Circulation.

[44] Skinner AC, Steiner MJ, Henderson FW, Perrin EM (2010). Multiple markers of inflammation and weight status: Cross-sectional analyses throughout childhood. Pediatrics.

